# Clinical and Molecular Heterogeneity Underlying Monogenic Causes of Pediatric Diabetes Associated to Brain Developmental Disorders

**DOI:** 10.1111/cge.70066

**Published:** 2025-09-19

**Authors:** Gabriele Di Pasquale, Camilla Valsecchi, Giulia Marie Smylie, Vincenzo Salpietro, Gian Vincenzo Zuccotti, Maurizio Delvecchio, Chiara Mameli

**Affiliations:** ^1^ Department of Biotechnological and Applied Clinical Sciences University of L'Aquila L'Aquila Italy; ^2^ Department of Pediatrics V. Buzzi Children's Hospital Milan Italy; ^3^ Department of Pediatrics Legnano Hospital Legnano Italy; ^4^ Department of Biomedical and Clinical Sciences University of Milan Milan Italy

**Keywords:** diabetes mellitus, molecular pathway, monogenic diabetes mellitus, neurodevelopmental disorders, next‐generation sequencing

## Abstract

Monogenic Diabetes Mellitus refers to heterogeneous forms of diabetes mellitus (DM) caused by a single gene pathogenic variant. Neurodevelopmental disorders (NDDs) are clinically and molecularly heterogeneous conditions characterized by an impairment of the nervous system development and/or function, with a wide clinical spectrum of variability. Over the last decade, Next Generation Sequencing (NGS) approaches have played a crucial role in the discovery of many monogenic causes underlying both NDDs and diabetes. In this systematic review, we aim to overview novel and emerging monogenic diseases presenting with pediatric diabetes and concomitant NDDs. The literature search was run in PubMed and Embase with a set of appropriate keywords. We examined 26 articles. Pathogenic variants have been classified according to the age of diabetes onset. In‐depth analysis has been conducted for the selected papers, focusing on clinical description and molecular implications for a definite disease‐causing gene. Interesting papers have revealed in recent years the occurrence of potential shared disease mechanisms underlying glucose and insulin metabolism and brain development and function. The broad clinical and molecular spectrum of DM‐associated NDDs highlights the importance of a comprehensive and multidisciplinary management of these emerging clinical conditions and the increasingly crucial role of appropriate therapeutic approaches.

## Graphical abstract

A growing number of genetic variants linking non‐autoimmune diabetes to NDDs across different ages offer key insights about a common background of these phenotypes. These findings call for multidisciplinary approaches to care that integrate metabolic and neurological management in affected children.

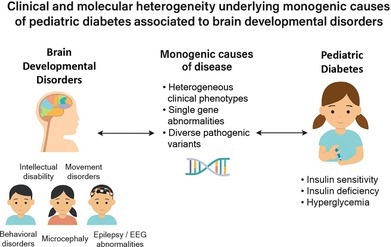

## 1 Introduction

Significant progress has been made over the past two decades in understanding the genetic bases of different subtypes of diabetes mellitus and neurodevelopmental disorders (NDDs) [1, 2]. Advances have been particularly prominent in the identification of genes predisposing to both NDDs and diabetes with onset in the neonatal period, early childhood, adolescence, as well as young adulthood [3]. The genetic background is crucial in understanding the pathophysiology of both conditions and could provide novel insights into their potential shared mechanisms.

In particular, strong links have been found between NDDs and neonatal diabetes mellitus (NDM), with KCNJ11 and ABCC8 being the most common genes involved [4]. *KCNJ11* encodes for ATP‐sensitive potassium channel subunit Kir6.2, which plays a key role in beta‐cell function. Carriers of *KCNJ11* pathogenic variants have been shown to present both NDM and broad neurological disorders, including hypotonia, intellectual disability (ID), epilepsy, and movement disorders, as the same pathogenic variant could lead to both metabolic and neurodevelopmental (NDD) impairment, probably resulting from expression of aberrant K_ATP_ channels in the central nervous system (CNS) [5]. Neuropsychiatric disorders like attention deficit hyperactivity disorder (ADHD), dyslexia, and autism spectrum disorders (ASDs) could occur as well. Moreover, subjects with *KCNJ11* pathogenic variants are often resistant to antiepileptic drugs but could benefit from sulfonylurea treatment, not only in terms of improved glycemic control but also in ameliorating NDD outcomes [6, 7]. *ABCC8* encodes for ATP‐binding cassette (ABC) transporter acting as a modulator of ATP‐sensitive potassium channels to insulin release. *ABCC8* patients often suffer from neurological impairment, varying from severe “DEND syndrome” with developmental delay, epilepsy, and NDM to milder presentations [8]. Even in *ABCC8* patients, it has been observed that sulfonylurea treatment could improve both glycemic and neurological outcomes, and long‐term neurological outcomes are better when treatment is initiated earlier [9].

In the last decade, Next Generation Sequencing (NGS), including whole exome sequencing (WES) and whole genome sequencing (WGS), enabled a comprehensive analysis of individual genetic makeup and allowed the identification of a large number of novel genes associated with Mendelian disease [2, 10, 11]. These studies also led to the understanding of the broad molecular heterogeneity associated with pediatric metabolic diseases and NDDs [11, 12].

The aim of this study is to provide an overview of novel and emerging monogenic diseases presenting with pediatric diabetes and concomitant NDDs, defining diagnostic and prognostic implications of certain disease‐causing genes as well as strategies in the therapeutic management of the children affected by these conditions.

## 2 Materials and Methods

This review used the PRISMA statement for systematic reviews. The literature search was launched on the 31st of March 2025 in PubMed and Embase. The keywords used were “neurodevelopmental disorders,” “intellectual disability,” “developmental delay,” “language delay,” “developmental regression,” “macrocephaly,” “microcephaly,” and “diabetes mellitus.” Non‐English language papers were excluded. We included retrospective studies, observational studies, and case reports published starting from 2013, when NGS started to be widely used in the diagnostic workup of patients suspected to have genetic disorders. Reviews, commentaries, editorials, and guidelines were excluded. Only articles describing patients diagnosed with monogenic NDDs and also presenting non–type 1 diabetes mellitus (within 25 years of age) were incorporated in this review. All pathogenic variants described before 2013 and all articles in which the exact gene variants were not reported have not been included in this project. Table S1 displays previously identified causes of NDDs and diabetes. The study has been registered in the PROSPERO database (ID: 1043330).

### 2.1 Data Extraction

**FIGURE 1:**
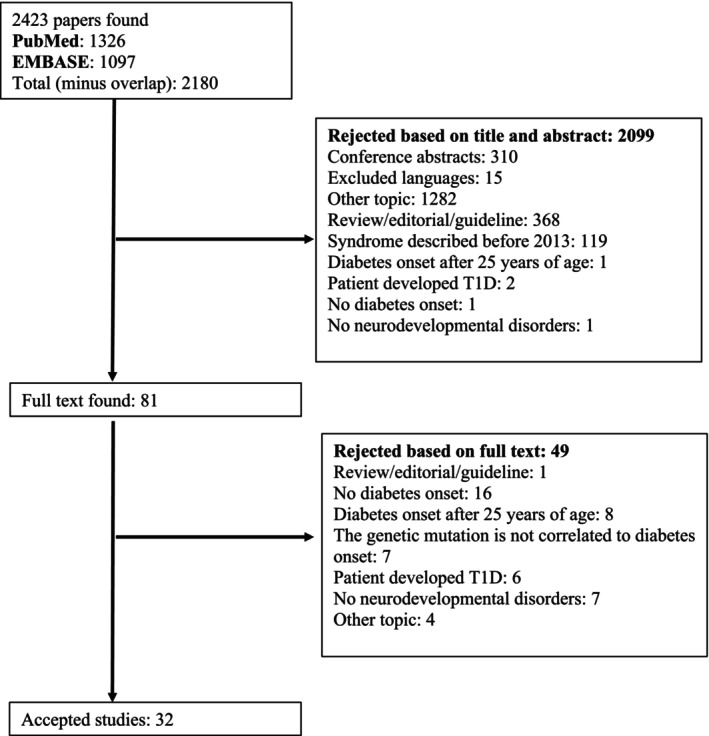
Flow diagram of study selection process.

Two authors (G.S. and C.V.) worked independently on the two online databases. The search retrieved 2423 papers. They screened all records and excluded 243 duplicates. The remaining 2180 records were screened by title and abstract, and 2099 were excluded. Eighty‐one full texts of potentially eligible papers were retrieved for evaluation. Disagreements between authors were resolved by discussion and consensus, with the overview of the senior authors. At the end of the selection process, 26 manuscripts were selected for this review (Figure 1).

This article is based on previously conducted studies and does not contain new studies with human participants or animals performed by any of the authors.

### 2.2 Outcomes

The main outcome is the description of clinical features of children, adolescents, and young adults with genetically determined NDDs who developed non‐type 1 diabetes mellitus before 25 years of age.

Informations were extracted from each manuscript and summarized as (1) participants' features (age, sex, and ethnicity); (2) pathogenic variant; (3) diabetes pattern; (4) neurologic features; (5) other clinical features.

### 2.3 Data Analysis

Data were extracted from the included papers and summarized using a narrative analysis. The results of each paper are displayed in Tables 1 and 2. Data were synthesized thematically.

**TABLE 1:** Clinical and genetic features in individuals with diabetes mellitus and neurodevelopmental disorders according to age at onset (Panel A: Neonatal onset; Panel B: Childhood onset; Panel C: Adolescence onset: Panel D: Young adulthood onset).

Gene	Age	Sex	Ethicity	Pathogenic variant	Zygosity	Diabetes features	Neurological features	Other features	Main tissue expression	References
*Panel A*										
PDIA6	Died (10 m)	M	Middle East	c.703del; p.(Val235fs)	Homozygous	Insulin‐dependent diabetes	Microcephaly, global ND	IUGR, PCKD, liver fibrosis, ATD, dysmorphic features	Brain, pancreas, kidney, liver	Al‐Fadhli et al. [13]
	Died (18 m)	M	Middle East	c.947dup; p.(Tyr316*)	Homozygous	Insulin‐dependent diabetes	Microcephaly, global ND, hypotonia	IUGR, PCKD, liver fibrosis		De Franco et al. [14]
YIPF5	5 y	M	Turkey	c.542C>T; p.(Ala181Val)	Homozygous	Insulin‐dependent diabetes	Microcephaly, epilepsy	N.R.	Brain, pancreas, kidney, liver	De Franco et al. [15]
	Died (1.3 y)	M	India	c.317_319del; p.(Lys106del)	Homozygous	Insulin‐dependent diabetes	Microcephaly, epilepsy	N.R.		De Franco et al. [15]
	21 y	F	Turkey	c.293T>G; p.(Ile98Ser)	Homozygous	Insulin‐dependent diabetes	Microcephaly, epilepsy, ND, no speak	N.R.		De Franco et al. [15]
	15 y	F	Turkey	c.293T>G; p.(Ile98Ser)	Homozygous	Insulin‐dependent diabetes	Microcephaly, epilepsy, ND, no speak	N.R.		De Franco et al. [15]
	5.5 y	F	Turkey	c.652T>A; p.(Trp218Arg)	Homozygous	Insulin‐dependent diabetes	Microcephaly, epilepsy, ND, no speak	N.R.		De Franco et al. [15]
	6 m	M	India	c.290G>T; p.(Gly97Val)	Homozygous	Insulin‐dependent diabetes	Microcephaly, epilepsy, ND	N.R.		De Franco et al. [15]
TARS2	Died (4 m)	F	Iran	c.980G>A, p.(Arg327Gln)	Homozygous	Insulin‐dependent diabetes	Epilepsy	LBW	Brain, pancreas, muscle	Donis et al. [16]
	Died (10 m)	F	Turkey	c.980G>A, p.(Arg327Gln)	Homozygous	Insulin‐dependent diabetes	ND	LBW, feeding difficulties		Donis et al. [16]
	Died (3 m)	M	India	c.980G>A, p.(Arg327Gln)	Homozygous	Insulin‐dependent diabetes	ND, epilepsy	LBW, hypoparatyroidism		Donis et al. [16]
	Died (19 m)	M	England	c.980G>A, p.(Arg327Gln)	Homozygous	Insulin‐dependent diabetes	ND, epilepsy	LBW, renal tubulopathy		Donis et al. [16]
*Panel B*										
SMPD4	11 y	M	N.R.	c.1188+2dup + c.2124_2125del, p.(Phe709*)	Compound heterozygous	Insulin‐dependent diabetes (no Ab)‐DKA	Microcephaly, global ND, epilepsy, hypotonia	IUGR, ventilator‐dependent	Brain, pancreas, bone, lung	Aoki et al. [17]
	45 y	F	N.R.	c.2431C>T, p.(Arg811Cys)	Homozygous	Insulin‐dependent diabetes (no Ab)	Microcephaly, ID, ataxic gait	Short stature		Smits et al. [18]
	43 y	F	N.R.	c.2431C>T, p.(Arg811Cys)	Homozygous	Insulin‐dependent diabetes (no Ab)	Microcephaly, ID, ataxic gait	Short stature		Smits et al. [18]
	41 y	F	N.R.	c.2431C>T, p.(Arg811Cys)	Homozygous	Insulin‐dependent diabetes (no Ab)	Microcephaly, ID, ataxic gait	Short stature		Smits et al. [18]
	15 y	M	N.R.	c.940delT, p.(Ser314Profs*60)	Homozygous	Insulin‐dependent diabetes (no Ab)	Microcephaly, ND, hypotonia	Joint contractures		Smits et al. [18]
	5 y	M	N.R.	c.370G>T; p.(Glu124*)	Homozygous	Insulin‐dependent diabetes (no Ab)	Microcephaly, ND, epilepsy, hypertonia	Joint contractures, Respiratory failure, RD		Smits et al. [18]
	4 y	M	N.R.	c.370G>T; p.(Glu124*)	Homozygous	Insulin‐dependent diabetes (no Ab)	Microcephaly, ND, epilepsy, hypertonia	Joint contractures, Respiratory failure		Smits et al. [18]
NBAS	34 y	M	Japan	c.5741G>A; p.(Arg1914His) + c.6433‐2A>G	Compound heterozygous	Insulin stopped; C‐peptide partly preserved (no Ab)	ID, epilepsy	LBW, short stature, joint contractures, Acute liver failure, Pelger‐Huët anomaly	Brain, pancreas, leukocytes, liver	Suzuki et al. [19]
	27 y	F	Hispanic	c.5741G>A; p.(Arg1914His) + c.17C>A (p.Ser6*)	Compound heterozygous	Insulin‐dependent diabetes (no Ab)	ND, hypertonia	LBW, short stature, dysmorphism, SOPH syndrome		Lacassie et al. [20]
*Panel C*										
LMNA	17 y	F	Hispanic	c.1634G>A; p.(Arg545His)	Homozygous	Insulin‐dependent diabetes (no Ab)‐DKA	Global ND	Hyper‐TG, hepatic steatosis, low total body fat	Widely expressed	Patni et al. [21]
	19 y	F	Hispanic	c.1634G>A; p.(Arg545His)	Homozygous	Insulin‐dependent diabetes (no Ab)	ID, speech delay	Hepatic steatosis, low total body fat, cataract		Patni et al. [21]
TRMT10A	26 y	F	Moroccan	c.379G>A; p.(Arg127Stop)	Homozygous	Insulin‐dependent diabetes (no Ab)	Microcephaly, ID, epilepsy	Short stature, dysmorphism, osteoporosis	Widely expressed: mostly brain and pancreas	Igoillo‐Esteve et al. [22]
	19 y	F	Moroccan	c.379G>A; p.(Arg127Stop)	Homozygous	Insulin‐dependent diabetes (no Ab)	Microcephaly, ID	Short stature		Igoillo‐Esteve et al. [22]
	21 y	M	Moroccan	c.379G>A; p.(Arg127Stop)	Homozygous	Insulin‐dependent diabetes (no Ab)	Microcephaly, ID	Short stature		Igoillo‐Esteve et al. [22]
	N.R	N.R.	Chinese	c.496‐1G>A	Homozygous	Non‐Insulin dependent diabetes (no Ab)‐Metformin	Microcephaly, ID	Short stature		Lin et al. [23]
	15 y	M	Israel	p.(Gly206Arg)	Homozygous	Insulin‐dependent diabetes (no Ab)	Microcephaly, ND	LBW, short stature, dysmorphism, obesity		Brener et al. [24]
	17 y	F	Turkey	c.379C>T, p.(Arg127*)	Homozygous	Non‐Insulin dependent diabetes (no Ab)‐ Metformin	Microcephaly, ID, epilepsy	LBW, short stature, dysmorphism, ovarian failure		Şıklar et al. [25]
	24 y	F	Caucasian	c.79G>T; p.(Glu27Ter)	Homozygous	Insulin‐dependent diabetes (no Ab)	Microcephaly, ID, epilepsy	Proliferative retinopathy		Yew et al. [26]
	28 y	M	Caucasian	c.79G>T; p.(Glu27Ter)	Homozygous	Non‐Insulin dependent diabetes (no Ab)‐Metformin	Microcephaly, ID	N.R.		Yew et al. [26]
	27 y	M	Turkey	C.23dup; p.(Phe9fs)	N.R.	Type 2 diabetes	Microcephaly, ID, epilepsy	LBW, short stature		Firdevs Ezgi Uçan Tokuç et al. [27]
	11y	F	Jewish	c.616G>A, p.(Gly206Arg)	Homozygous	Insulin‐dependent diabetes; weakly positive Ab (anti‐ICA: 76.9 IU/mL; anti‐GAD 7.4 IU/mL)	ND	LBW, IUGR, Hypoplastic kidney		Stern et al. [28]
MANF	14 y	F	Turkey	c.82_94del, p.(Leu28Thrfs*33)	Homozygous	Insulin‐dependent diabetes (no Ab)	Microcephaly, ND	Short stature, deafness	Widely expressed: mostly brain, liver and pancreas	Montaser et al. [29]
	27 y	F	Turkey	c.103+1G>T	Homozygous	Insulin‐dependent diabetes (no Ab)	Microcephaly, ND	Short stature, deafness		Montaser et al. [30]
PTRH2	12 y	F	India	c.127dupA; p.(Ser43LysfsTer11)	Homozygous	Insulin‐dependent diabetes (no Ab)‐ DKA	ID, SHL	N.R.	Muscle, Brain, Pancreas	Parida et al. [30]
	15 y	M	Tunisia	c.254A>C; p.(Glu85Pro)	Homozygous	Insulin‐dependent diabetes (no Ab)	Hypotonia, ND, SHL	N.R.		Sylvie Picker‐Minh et al. [31]
PPP1R15B	28 y	M	Algeria	c.1972C>T; p.(Arg658Cys)	Homozygous	Insulin‐dependent diabetes (no Ab)	Microcephaly, ID, SHL	Short stature	Liver, Kidney, Pancreas, Brain	Abdulkarim et al. [32]
IARS	19 y	F	Japan	c.760C>T + c.1310C>T	Compound heterozygous	Insulin‐dependent diabetes (no Ab)	Microcephaly, ID, epilepsy, SHL	IUGR	Widely expressed: mostly muscle, brain, pancreas	Kopajtich et al. [33]
DNAJC3	15 y	M	Turkey	c.393+2T>G	Homozygous	Insulin‐dependent diabetes (no Ab)	Microcephaly, ND, epilepsy, SHL	Short stature	Widely expressed: mostly skin, liver, brain, pancreas	Alev Ozon et al. [34]
	13 y	F	Turkey	c.393+2T>G	Homozygous	Non‐Insulin dependent diabetes (no Ab)‐diet	Microcephaly, ND, epilepsy, motor disorders, SHL	Short stature		Alev Ozon et al. [34]
	17 y	M	Middle East	c.1177C>T, p.(Arg393Ter)	Homozygous	Insulin‐dependent diabetes (no Ab)	ID, SHL	Short stature		Alwatban et al. [35]
	28 y	M	Middle East	c.1177C>T, p.(Arg393Ter)	Homozygous	Insulin‐dependent diabetes (no Ab)	ID, SHL	Short stature		Alwatban et al. [35]
	30 y	F	France	c.1036C>T + c.1A>G	Compound heterozygous	Non‐Insulin dependent diabetes (no Ab)‐GLP‐1 RA	Microcephaly, SHL	Short stature		Lytrivi et al. [36]
	18 y	M	Algeria	c.1177C>T, p.(Arg393*)	Homozygous	Insulin‐dependent diabetes (no Ab)	SHL	Short stature		Lytrivi et al. [36]
*Panel D*										
CPE	20 y	F	Sudan	c.76_98del	Homozygous	T2D	ID	Obesity, hypogonadism	Brain, pancreas, enteroendocrine cells	Alsters et al. [37]
CARS	34 y	M	Caucasian	c.2061dup; p.(Ser688Glnfs*2)	Homozygous	T2D	Microcephaly, ID, peripheral neuropathy, hypotonia	IUGR, short stature, dysmorphism, osteoporosis	Widely expressed; main in brain, pancreas, lung	Kuo et al. [38]
	35 y	F	Dutch	c.1022G>A + c.1138C>T	Compound heterozygous	Diabetes (not specified)	Microcephaly, ND, motor disorder	IUGR, steatosis		Kuo et al. [38]

Abbreviations: Ab, antibody; ATD, Asphyxiating thoracic dystrophy; DKA, Diabetic ketoacidosis; GAD, glutamic acid decarboxylase antibodies; GLP‐1 RA, Glucagon‐like peptide‐1 receptor agonists; Hyper‐TG, Hypertrygliglyceridemia; ICA, islet cell antibodies; ID, Intellectual disability; IUGR, Intra‐uterine growth restriction; LBW, Low birth weight; m, months; N.R., not reported; ND, Neurodevelopmental delay; PCKD, Polycystic kidney disease; RD, Retinal dystrophy; SHL, Sensorineural hearing loss; T2D, Type 2 diabetes; y, years.

**TABLE 2:** Diabetes characteristics.

	References	Gene	Age at diagnosis of diabetes	Tested diabetes autoantibodies	C‐peptide levels (on blood, if not differently specified)	Insulin dose (U/kg/d)	Hemoglobin A1c
Neonatal Diabetes	De Franco et al. [4]	YIPF5	Pt 1: 9 w Pt 2: 15 w Pt 3: 15 m Pt 4: 8.5 m Pt 5: 4 w Pt 6: 23 w	N/A	Pt 1: 99 pmol/L Pt 2: N/A Pt 3: 95 pmol/L Pt 4: 147 pmol/L Pt 5: 46 pmol/L Pt 6: N/A	Pt 1: 0.73 Pt 2: 1.7 Pt 3: 0.87 Pt 4: 0.8 Pt 5: 0.77 Pt 6: 0.9	Pt 1: 8.7% Pt 2: N/A Pt 3: 8.7% Pt 4: 8.7% Pt 5: 10.7% Pt 6: 14.8%
	De Franco et al. [14]	PDIA6	8 m	N/A	N/A	N/A	N/A
	Fatima M. Al‐Fadhli et al. [13]	PDIA6	Various episodes of hyperglycemia starting from the 2nd day of life	N/A	N/A	N/A	N/A
	Russel Donis et al. [16]	TARS2	Pt 1: 1 w Pt 2: 1 w Pt 3: 1 d Pt 4: 1 y	N/A	N/A	Pt 1: N/A Pt 2: 0.3 Pt 3: 1.1 Pt 4: 0.3	N/A
Childhood‐onset diabetes	Shigeru Suzuki et al. [19]	NBAS	6 y	ICA Ab: negative at 19 y	Urine C‐peptide levels of 29.6 μg/day at 20 y	Insulin therapy could temporarily be stopped over 1 year from onset	9.1%
	Lacassie et al. [20]	NBAS	11 y	N/A	N/A	N/A	7.0%
	Smits et al. [18]	SMPD4	Pt 1: 13–14 y Pt 2: 13–14 y Pt 3: 13–14 y Pt 4: 15 y Pt 5: T1D at 3 y Pt 6: T1D at 4 y	Pt 1–5: N/A Pt 6: negative IAA Ab	N/A	Pt 1–5: N/A Pt 6: 0.8	Pt 1–5: N/A Pt 6: 7.4%
	Aoki et al. [17]	SMPD4	6 y	Negative ICA Ab, IA‐2 Ab, GAD Ab, IAA Ab	N/A	N/A	Pt 2: 9.6%
Adolescent onset diabetes	Nivedita Patni et al. [21]	LMNA	Pt 1: 11 y Pt 2: 16 y	Pt 1: negative ICA Ab, IA‐2 Ab, GAD Ab, IAA Ab Pt 2: N/A	Pt 1: N/A Pt 2: 3.95 ng/mL	Pt 1: ~3.8 Pt 2: N/A	Pt 1: 7.2% Pt 2: 6.7%
	Hossam Montaser et al. [29]	MANF	Pt 1: 10 y Pt 2: 17 y	Pt 1–2: negative ICA Ab	Pt 1: 240 pmol/L 4 y after diagnosis Pt 2: 330 pmol/L 4 years after diagnosis	N/A	N/A
	Robert Kopajtich et al. [33]	IARS	16 y	N/A	N/A	N/A	N/A
	Sylvie Picker‐Minh et al. [31]	PTRH2	Pt 1: N/A	N/A	N/A	N/A	Pt 1: 11.5%
	Parida et al. [30]	PTRH2	12 y	N/A	N/A	N/A	12.8%
	Abdulkarim et al. [32]	PPP1R5B	Pt 1: 15 y Pt 2: 28 y	Pt 1: negative ICA Ab, IA‐2 Ab, GAD Ab Pt 2: N/A	Pt 1: 3.96 nmol/L Pt 2: N/A	Pt 1: 0.5 at 28 y Pt 2: 0.7	Pt 1: 13% Pt 2: N/A
	Alev Ozon et al. [34]	DNAJC3	Pt 1: 12 y Pt 2: 13 y	Pt 1: negative ICA Ab, IA‐2 Ab, GAD Ab Pt 2: ICA Ab, GAD Ab	Pt 1: 0.78 nmol/L Pt 2: N/A	Pt 1: N/A	Pt 1: 7.1% Pt 2: 5.9%
	Lytrivi et al. [36]	DNAJC3	Pt 1: 12 y Pt 2: 16 y	Pt 1: negative IA‐2 Ab, GAD Ab Pt 2: N/A	Pt 1: 314 pM at 19 y Pt 2: N/A	Pt 2: N/A	Pt 1: 7.2%–8.5% Pt 2: 8.5%
	Saud Alwatban wt al. [35]	DNAJC3	Pt 1: 11 y Pt 2: 14 y	Pt 1: negative ICA Ab, IAA Ab, GAD Ab Pt 2: N/A	Pt 1: in range Pt 2: N/A	Pt 1: 0.5 (insulin started 3 y later) Pt 2: N/A	Pt 1: 7.6% Pt 2: N/A
	Avivit Brener et al. [24]	TMRT10A	15 y	Negative pancreatic autoantibodies	N/A	2, 3 y after diagnosis	15.6%

	Zeynep Şıklar et al. [25]	TMRT10A	11 y	Negative ICA Ab, IAA Ab, GAD Ab	1.29 ng/mL		7.4%
	Stern et al. [28]	TMRT10A	11 y	Positive ICA Ab and weakly positive GAD Ab	2.47 ng/mL 2 y after diagnosis	0.4, 2 y after diagnosis	9.9%
	Lin et al. [23]	TMRT10A	N/A	Negative ICA Ab, IAA Ab, GAD Ab, IA‐2 Ab, ZnT8 Ab	Well preserved		14.4%
	Igoillo‐Esteve et al. [22]	TMRT10A	Pt 1: 22 y Pt 2: 19 y Pt 3: 14 y	Pt 1–3: negative ICA Ab, IAA Ab, GAD AB, IA‐2 Ab	Pt 1–3: in range 20 y after diagnosis	Pt 1–3: 0.4–1.2	Pt 1: 11.3% Pt 2: 13.2% Pt 3: 11.1%
	Yew et al. [26]	TMRT10A	Pt 1: 24 y Pt 2: 28 y	Pt 1: negative GAD Ab and IA‐2 Ab. Pt 2: N/A	Pt 1: 540 pmol/L 8 y after diagnosis Pt 2: 1000 pmol/L	Pt 1: 1–1.2	Pt 1: 15.1% Pt 2: 7.6%
Firdevs Ezgi Uçan Tokuç et al. [27]	TMRT10A	T2D at 15 y	N/A	N/A		N/A
Young adult‐onset diabetes	Alsters et al. [37]	CPE	T2D at 15 y	N/A	N/A		12.6%
	Molly Kuo et al. [38]	CARS	Pt 1: T2D at 24 y Pt 2: 30 y	N/A	N/A	N/A	N/A

Abbreviations: d, days; DM, diabetes mellitus; GAD, Glutamic Acid Decarboxylase Antibodies; IA‐2, Islet Antigen‐2 Autoantibodies; IAA, Insulin Autoantibodies; ICA, Islet Cell Antibodies; m, months; N/A, not available; Pt, Patient; T1D, Type 1 diabetes; T2D, Type 2 diabetes; w, weeks; y, years; ZnT8, Zinc Transporter 8 Autoantibodies.

Techniques used for genetic analysis and evaluation of protein expression in human tissues are summarized in Table 3.

**TABLE 3:** Techniques used for genetic analysis and evaluation of protein expression in human tissues.

	References	Gene	Genetic analysis	Other analysis	Tissue expression in humans
Neonatal diabetes	De Franco et al. [4]	YIPF5	NGS; Sanger sequencing		qPCR; ISH
	De Franco et al. [14]	PDIA6	WGS; tNGS		
	Fatima M. Al‐Fadhli et al. [13]	PDIA6	WES; Sanger sequencing		RT‐PCR
	Russel Donis et al. [16]	TARS2	WGS; tNGS		
Childhood‐onset diabetes	Shigeru Suzuki et al. [19]	NBAS	NGS; Sanger sequencing	Western blots; RNA analysis	RT‐PCR
	Lacassie et al. [20]	NBAS	WES		
	Smits et al. [18]	SMPD4	ES		RT‐PCR
	Aoki et al. [17]	SMPD4	ES; Sanger sequencing	Minigene splicing assay on RNA splicing	RT‐PCR
Adolescent onset diabetes	Nivedita Patni et al. [21]	LMNA	WES; Sanger sequencing		
	Hossam Montaser et al. [29]	MANF	NGS; Sanger sequencing		RT‐PCR
	Robert Kopajtich et al. [33]	IARS	WES; Sanger sequencing		Immunoblot analysis
	Sylvie Picker‐Minh et al. [31]	PTRH2	WES; Sanger sequencing	Western blot	RT‐PCR
	Parida et al. [30]	PTRH2	WES; Sanger sequencing	Chromosomal microarray	
	Abdulkarim et al. [32]	PPP1R5B	ES; Sanger sequencing	RFLP	
	Alev Ozon et al. [34]	DNAJC3	WES; Sanger sequencing		
	Lytrivi et al. [36]	DNAJC3	WES; Sanger sequencing	RFLP	qPCR
	Saud Alwatban et al. [35]	DNAJC3	WES		
	Avivit Brener et al. [24]	TMRT10A	NGS; WES; Sanger sequencing		
	Zeynep Şıklar et al. [25]	TMRT10A	tNGS		
	Stern et al. [28]	TMRT10A	WES	Microarray	
	Lin et al. [23]	TMRT10A	tNGS; ES; Sanger sequencing		
	Igoillo‐Esteve et al. [22]	TMRT10A	WES; Sanger sequencing	qRT‐PCR	RNA in situ hybridization
	Yew et al. [26]	TMRT10A	tNGS; Sanger sequencing	PCR	
	Firdevs Ezgi Uçan Tokuç et al. [27]	TMRT10A	NGS		
Young adult‐onset diabetes	Alsters et al. [37]	CPE	WES; Sanger sequencing		RT‐PCR
	Molly Kuo et al. [38]	CARS	NGS; ES; Sanger sequencing	Immunoblot analysis	

Abbreviations: ES, Exome sequencing; ISH, In Situ Hybridization; NGS, Next‐Generation Sequencing; PCR, Polymerase Chain Reaction; qPCR, quantitative PCR; qRT‐PCR, Quantitative real‐time PCR; RFLP PCR, Restriction Fragment Length Polymorphism PCR; RT‐PCR, Reverse Transcriptase‐PCR; tNGS, targeted NGS; WES, Whole‐Exome Sequencing; WGS, Whole‐Genome Sequencing.

## 3 Results

The 26 articles examined in this study provide significant insights into genetic variants associated with diabetes and NDDs. We classified these pathogenic variants according to the age of diabetes onset: neonatal diabetes (0–6 months), childhood diabetes (6 months–10 years), adolescent diabetes (11–18 years) and young adult diabetes (19–25 years). Clinical features for each gene are summarized in Table 1.

### 3.1 Neonatal Diabetes

Three new genes causing NDM and NDDs were described: pathogenic variants of *PDIA6* (MIM*611099), encoding a Protein Disulfide Isomerase Family A Member, were identified in two patients. Pathogenic variants in *TARS2* (MIM*612805), encoding a mitochondrial threonyl‐tRNA (Thr‐tRNA) synthetase, were found in three patients. Pathogenic variants of *YIPF5* (MIM*611483), encoding Yip1 Domain Family Member 5, were detected in six patients. Reported *YIPF5* variants were all homozygous, and affected children were born from consanguineous marriages in all families described. *PDIA6* pathogenic variants [13, 14] were described in two babies born at 32 weeks of gestation due to intrauterine growth retardation (IUGR); interestingly, both patients presented with microcephaly, dysmorphic features, polycystic kidney disease (PCKD), and developed neurological impairment and insulin‐dependent diabetes. *TARS2* pathogenic variants were identified in three unrelated probands with NDM and profound brain developmental disorders with or without drug‐resistant seizures; all died in the first year of life [16]. *YIPF5* pathogenic variants, instead, were associated with insulin‐dependent neonatal/early‐onset diabetes, severe microcephaly, severe epilepsy, and profound developmental delay [15].

### 3.2 Childhood‐Onset Diabetes

Childhood‐onset diabetes was diagnosed in children variably carrying pathogenic variants in (i) the *SMPD4* gene (MIM*610457), encoding Sphingomyelin Phosphodiesterase; (ii) the *NBAS* gene (MIM*608025), encoding for Biallelic Neuroblastoma Amplified Sequence. In total, eight patients with pathogenic variants of *SMPD4* were reported [17, 18], seven of whom developed non‐autoimmune insulin‐dependent diabetes during childhood/adolescence. The only patient that didn't develop diabetes was four years old at the last follow‐up visit. Pathogenic variants of this gene were also associated with other neurological comorbidities such as microcephaly, NDDs, and brain abnormalities.

Two patients were described with *NBAS* (Biallelic Neuroblastoma Amplified Sequence) pathogenic variants. The first was diagnosed with insulin‐dependent diabetes at 6 years of age, with progressive beta cell dysfunction; he achieved normal milestones during infancy but showed psychomotor regression, intellectual disability, and epilepsy during childhood. He also displayed severe short stature, dysmorphic features, and developed glaucoma, common variable immunodeficiency, autoimmune hemolytic anemia, and hepatic cirrhosis [19]. The latter had distinctive craniofacial features and macrocephaly, NDDs, hypertonia, and failure to thrive, as well as optic nerve atrophy. She was diagnosed with diabetes at the age of 11 years [20].

### 3.3 Adolescent Onset Diabetes

In recent years, a wide range of emerging genes have been implicated in adolescent‐onset diabetes mellitus and heterogeneous NDDs. These include *LMNA* (MIM*150330) encoding Lamin A/C, *TRMT10A* (MIM*616013) encoding the tRNA Methyltransferase 10A, *MANF* (MIM*601916) encoding Mesencephalic Astrocyte‐Derived Neurotrophic Factor, *PTRH2* (MIM*608625) encoding Peptidyl‐tRNA Hydrolase 2, *PPP1R15B* (MIM*613257) encoding the Protein Phosphatase 1 Regulatory Subunit 15B, *IARS* (MIM*600709) encoding the Isoleucyl‐tRNA Synthetase, and *DNAJC3* (MIM*601184) encoding DnaJ Heat Shock Protein Family Member C3.

Two subjects presenting with diabetes treated with insulin and metformin, an early‐onset ID, and other metabolic disorders with near‐generalized lipodystrophy, extreme hypertriglyceridemia, and liver steatosis resulted positive for *LMNA* pathogenic variants [21].

Ten cases of homozygous pathogenic variants in *TRMT10A* have been described in total; among these, seven developed diabetes mellitus during adolescence and three during adulthood [22, 23, 24, 25, 28, 39]. Two patients experienced frequent episodes of spontaneous hypoglycemia with onset in infancy [24, 25]. All patients had detectable C‐peptide at the onset of diabetes; some were treated only with metformin, while others were treated with insulin (with or without the addition of metformin). *TRMT10A* pathogenic variants were also associated with microcephaly, psychomotor delay, ID, epilepsy, short stature, dysmorphic facial features, and delayed puberty.

Pathogenic variants in the *MANF*, *PTRH2*, and *PPP1R15B* genes were associated with insulin‐dependent diabetes mellitus, intellectual disability, and sensorineural hearing impairment [29, 30, 32]. *MANF* and *PPP1R15B* variants were also linked to microcephaly and short stature, while *PTRH2* and *PPP1R15B* variants were associated with peripheral neuropathy and dental hypoplasia, respectively.

Pathogenic variants in *IARS* were described in three subjects [33], and one of them presented insulin‐dependent diabetes mellitus and severe (prenatal‐onset) growth retardation, hypotonia, microcephaly, ID, motor impairment, epilepsy, and sensorineural hearing loss.

Pathogenic variants of the *DNAJC3* were reported in six cases [34, 35, 36] and linked to various neurological disorders such as microcephaly, delayed psychomotor development, brain progressive neurodegeneration, polyneuropathy, ataxia, and hearing loss. Severe short stature, facial dysmorphisms, bone deformities, hypothyroidism, and diabetes mellitus were also associated with pathogenic variants in this gene. All patients developed diabetes during the second decade of life, and in four individuals, the onset of diabetes was preceded by spontaneous episodes of hypoglycemia during early childhood [34, 36].

### 3.4 Young Adult‐Onset Diabetes

Adult‐onset diabetes was diagnosed in three patients and associated with pathogenic variants in *CPE* (MIM*114855) encoding Carboxypeptidase E and *CARS* (MIM*123859) encoding a cysteinyl‐tRNA synthetase.

*CPE* pathogenic variant was detected in one patient with childhood‐onset obesity, type 2 diabetes, ID, and hypogonadotropic hypogonadism [37]. However, the authors did not clarify whether the hyperglycemia observed was a direct consequence of the pathogenic variant or secondary to obesity.

Furthermore, variants in *CARS* were reported in four patients, two of whom were diagnosed with diabetes [38]. All individuals exhibited a complex syndrome that included microcephaly, delayed psychomotor neurodevelopment, and brittle hair and nails.

## 4 Discussion

This systematic review study provides new insights into the genetic mechanisms linking non‐autoimmune diabetes with NDDs, showing how specific gene variants contribute to both conditions across different stages of development. Our analysis shows that diabetes may arise from shared molecular pathways that influence both pancreatic β‐cell function and brain development.

NDM, a rare monogenic diabetes form presenting within the first 6 months of life, is associated with pathogenic variants in more than 30 genes, typically leading to β‐cell loss or dysfunction [40]. Among the most studied are KCNJ11 and ABCC8, which encode subunits of the ATP‐sensitive potassium channel. Other genes, including PDIA6, YIPF5, and TARS2, are associated with syndromic NDM featuring severe NDDs and multisystem involvement. The tissue expression underlies the pleiomorphic phenotype. PDIA6 and YIPF5 encode ER‐resident proteins expressed mainly in the brain, pancreas, liver, and kidney and involved in stress response and protein folding.

Their dysfunction enhances ER stress and β‐cell apoptosis. Patients often present with microcephaly, IUGR, and early mortality [13, 14, 15]. TARS2, a mitochondrial gene involved in oxidative phosphorylation and mTORC1 signaling, is essential for energy homeostasis in β‐cells, neurons, and muscle cells. Pathogenic variants have been reported in infants with severe neurological symptoms, lactic acidosis, and diabetes, often resulting in early death [16].

Childhood‐onset syndromic diabetes has been linked to genes involved in diverse cellular functions. SMPD4, encoding a neutral sphingomyelinase, plays a critical role in lipid metabolism and signaling pathways [41] in the shared pathophysiology of metabolic and neurodevelopmental dysfunction. NBAS is involved in ER‐Golgi transport [42] and has been associated with liver failure syndromes and, more recently, non‐autoimmune diabetes [19, 20]. Compound heterozygous variants show variable expressivity, depending on the second allele [19, 43].

In adolescents, additional genes implicated in syndromic diabetes with NDDs include LMNA, TRMT10A, MANF, PTRH2, PPP1R15B, ASXL3, and DNAJC3. These genes are expressed more widely than the genes mentioned above, with a wide range of processes such as chromatin remodeling, ER stress responses, and nuclear architecture. LMNA variants, classically associated with progeroid syndromes, have been linked to a phenotype combining generalized lipodystrophy, NDDs, and diabetes, suggesting a role for nuclear envelope integrity in β‐cell function [21, 44, 45, 46, 47].

TRMT10A, a tRNA methyltransferase expressed in the brain and pancreatic islets, is associated with diabetes, microcephaly, intellectual disability, and epilepsy. In these patients, insulin sensitivity is highly variable and thus they are managed with insulin or metformin [22, 23, 25, 26, 27, 39]. ASXL3 mutations cause Bainbridge–Ropers syndrome, and recent reports describe co‐occurrence of insulin resistance and overt diabetes [48]. DNAJC3, involved in ER stress regulation, is associated with adolescent‐onset diabetes and NDDs, with some patients presenting episodes of hypoglycemia prior to disease onset [34, 36, 49, 50].

Variants in MANF, PTRH2, and PPP1R15B have also been linked to adolescent‐onset diabetes with coexisting intellectual disability, peripheral neuropathy, and sensorineural hearing loss, supporting the idea that shared molecular vulnerabilities underlie both metabolic and neurological dysfunction. As in carriers of MANF and PPP1R15B mutations, anti‐pancreatic antibodies have been reported; in the case of PTRH2, the antibodies titer is not reported, but the c‐peptide level is not suggestive of autoimmune diabetes [29, 30, 32].

Even if not included in the results section, data about the *MIA3* and *XRCC4* gene mutations suggest a possible role in diabetes and NDDs [51, 52]. The former was described in a large consanguineous Turkish family with four affected siblings with insulin‐dependent diabetes, mild ID, primary obesity, dentinogenesis imperfecta, short stature, hearing loss, and skeletal abnormalities. *MIA3* encodes a key mediator at the ER which interacts with *CTAGE5*—a protein essential for insulin secretion [53, 54]. The latter was described in a single case with microcephaly, NDDs, and non‐autoimmune diabetes, suggesting a link between genomic instability and β‐cell dysfunction. We did not include these genes in the results section because of the absence of other families with the same mutated genes that could confirm the findings.

An important question emerges: what is the contribution of dysglycemia—particularly early hyper‐ or hypoglycemia—to the neurodevelopmental outcomes observed in these patients? It is well known that glycemic extremes can harm neuronal integrity and that neonates of diabetic mothers may develop structural brain abnormalities. Neonatal hypoglycemia can also cause permanent brain damage [55].

Nevertheless, our data suggest that in most cases, the neurodevelopmental phenotype is primarily genetically determined in consideration of the tissue expression. Support for this statement comes from data on KCNJ11 and ABCC8. They are actionable genes, and sulfonylureas treatment improves not only glycemic control but also neurological symptoms, supporting the hypothesis that the underlying genetic defect plays a greater role than the glycemic environment in determining clinical outcomes.

On the other hand, this view is supported by observations in HNF4A‐MODY, which is frequently associated with neonatal hypoglycemia but does not lead to neurodevelopmental impairment later in life. While we cannot exclude that in certain cases, glycemic excursions may amplify neurological damage—especially when occurring during vulnerable developmental windows—the current evidence favors a primary role for the genetic lesion in driving both endocrine and neurological features.

The etiology of diabetes deserves a comment. It is possible that the described genes led to NDDs, and that the patient also, by coincidence, had the polygenic form of diabetes. Table 2 displays diabetes features to clarify the etiology, according to our previous papers and appropriate literature [56, 57, 58]. We believe there are sufficient data to rule out an autoimmune origin of the hyperglycemia. In infants with NDM, autoantibodies have not been reported, as this is, by definition, a monogenic form of diabetes. Only one carrier of TRMT10A [28] showed positive ICA and low‐titer anti‐GAD antibodies, but the c‐peptide level rules out beta‐cell deficiency, supporting the hypothesis of a different mechanism leading to hyperglycemia.

## 5 Conclusion

This review underscores the growing number of genetic variants linking non‐autoimmune diabetes to NDDs across different ages. Identifying age‐specific gene associations—from neonatal to adult onset—offers key insights into how temporally regulated gene expression influences both metabolism and brain development. Age‐dependent genotype–phenotype correlations underscore the pleiotropic and evolving nature of these disorders. These findings call for multidisciplinary approaches to care that integrate metabolic and neurological management in affected children.

Advances in NGS technique have uncovered novel monogenic disorders converging on shared molecular pathways affecting insulin regulation and neurodevelopment. Classifying gene–phenotype relationships by age of onset may support age‐tailored diagnostic and therapeutic strategies. The marked clinical and molecular heterogeneity of these syndromic forms underscores the urgency of comprehensive care and the development of targeted therapies that address the overlapping mechanisms between pancreatic and neurological dysfunction.

## Author Contributions

**Gabriele Di Pasquale:** writing – original draft, resources. **Camilla Valsecchi:** writing – original draft, Data curation. **Giulia Marie Smylie:** writing – original draft, data curation. **Vincenzo Salpietro Damiano:** validation, supervision. **Gian Vincenzo Zuccotti:** validation, supervision. **Maurizio Delvecchio:** writing – review and editing, conceptualization, project administration. **Chiara Mameli:** writing – review and editing, conceptualization, data curation.

## Conflicts of Interest

The authors declare no conflicts of interest.

## Peer Review

The peer review history for this article is available at https://www.webofscience.com/api/gateway/wos/peer‐review/10.1111/cge.70066.

## Supporting information


**Table S1:** Causes of genetic shared etiology of neurodevelopmental disorders (NDDs) and diabetes identified before 2013.

## Data Availability

Data sharing not applicable to this article as no datasets were generated or analyzed during the current study.
