# Supplementary material for: Clinical and Molecular Heterogeneity Underlying Monogenic Causes of Pediatric Diabetes Associated to Brain Developmental Disorders
Source: Clin Genet. 2025 Sep 19;108(5):495–510. doi: 10.1111/cge.70066 (PMC12501751; doi:10.1111/cge.70066)
Supplement: Supplementary file 1 — Table S1: Causes of genetic shared etiology of neurodevelopmental disorders (NDDs) and diabetes identified before 2013. [file CGE-108-495-s001.docx]

| **GENES** | **PHENOTYPE** |
| --- | --- |
| **KCNJ11/ ABCC8** | low birth weight, transient or permanent neonatal diabetes mellitus, DEND syndrome (Developmental delay, Epilepsy, and Neonatal Diabetes), muscle weakness and hypotonia |
| **PTF1A:** | low birth weight, neonatal diabetes mellitus, pancreatic agenesis/hypoplasia, exocrine pancreas insufficiency, cerebellar agenesis, neurodevelopmental delay, epilepsy, optic nerve hypoplasia, arm and legs flexion contractures or abnormal movements, dysmorphic features |
| **NEUROD1** | biallelic mutations of NEUROD1 are associated with low birth weight, permanent neonatal diabetes and a consistent pattern of neurological abnormalities including cerebellar hypoplasia, developmental delay, learning difficulties, sensorineural deafness and visual impairment. Heterozygous mutations are instead linked to the development of maturity-onset diabetes of the young (MODY) and late-onset diabetes |
| **CNOT1** | low birth weight, neonatal diabetes mellitus, pancreatic and gallbladder agenesis, exocrine pancreas insufficiency, holoprosencephaly, dysmorphic features |
| **NKX2-2** | low birth weight, neonatal diabetes mellitus, severe obesity, CNS abnormalities, developmental delay |
| **MNX1** | low birth weight, neonatal diabetes mellitus, developmental delay, hypotonia, cortical blindness, impaired visual tracking, hearing impairment, hypoplastic lungs, sacral agenesis, high imperforate anus |
| **PAX6** | neonatal diabetes mellitus, microcephaly, CNS anomalies, severe developmental delay, bilateral microphthalmia, panhypopituitarism, choanal atresia, renal dysplasia, cryptorchidism and micropenis |
| **IER3IP1** | neonatal diabetes, microcephaly, epilepsy and CNS anomalies |
| **EIF2S3** | neonatal diabetes, MEHMO syndrome (Mental retardation, Epileptic seizures, Hypogonadism, Microcephaly, Obesity), demyelinating and cerebral atrophy on brain MRI, dysmorphic features |
| **DCAF17** | Woodhouse-Sakati syndrome symptoms such as hypogonadism, diabetes (adolescent to young adult onset), mental retardation, alopecia, extrapyramidal findings, sensorineural hearing loss, hypothyroidism, dysmorphic features |
| **WFS1** | biallelic mutations of WFS1 cause ‘classic’ Wolfram syndrome (diabetes and optic atrophy before age of 16 years, sensorineural hearing impairment, cerebellar ataxia, autonomic dysfunction, psychiatric disease, urinary tract problems, central diabetes insipidus, hypogonadism and growth retardation) while heterozygous pathogenic variants may cause Wolfram syndrome-like disease (incomplete phenotype). |

**Supplementary table.** Causes of genetic shared etiology of neurodevelopmental disorders (NDDs) and diabetes identified before 2013.
